# Supplementary material for: Causal interplay between lactose intolerance and gut microbiota: a combined bidirectional Mendelian randomization and in vivo validation study
Source: Front Nutr. 2026 Jun 1;13:1803337. doi: 10.3389/fnut.2026.1803337 (PMC13265576; doi:10.3389/fnut.2026.1803337)
Supplement: Supplementary file 1 [file Data_Sheet_1.zip › supplementary materials/Forward/forest plot/ebi-a-GCST90027694.finngen_R12_E4_LACTONAS.pdf]

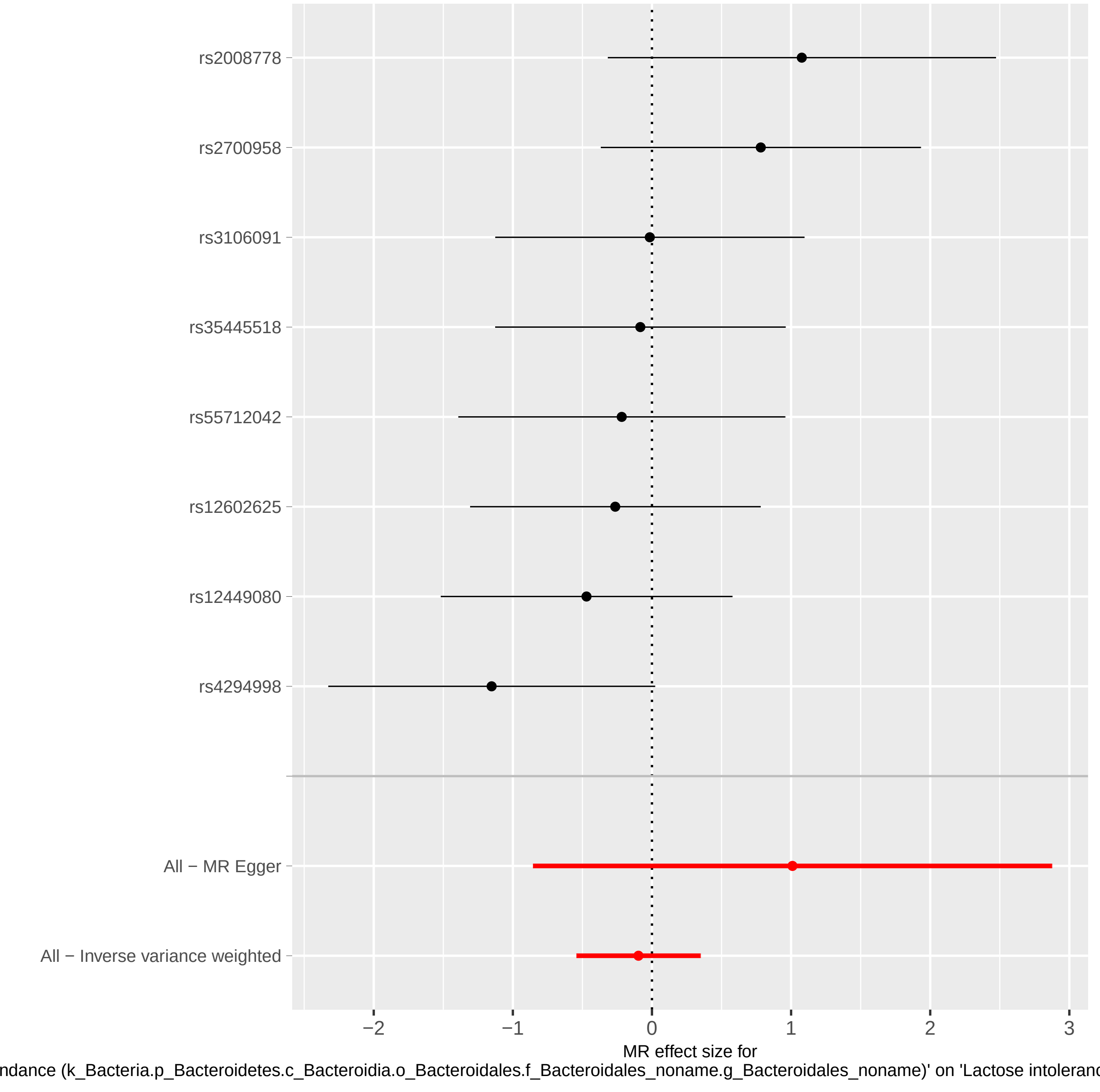
rs2008778
rs2700958
rs3106091
rs35445518
rs55712042
rs12602625
rs12449080
rs4294998
All − MR Egger
All − Inverse variance weighted
−2
−1
0
1
2
3
MR effect size for
ndance (k_Bacteria.p_Bacteroidetes.c_Bacteroidia.o_Bacteroidales.f_Bacteroidales_noname.g_Bacteroidales_noname)' on 'Lactose intoleranc
